# The Effect of Actives in Desensitizing and Conventional Mouth Rinses Against Dentin Erosive Wear

**DOI:** 10.1590/0103-6440202405500

**Published:** 2024-10-28

**Authors:** Diana Roberta Pereira Grandizoli, Letícia Oba Sakae, Ana Luísa Meira Renzo, Sávio José Cardoso Bezerra, Samira Helena Niemeyer, Taís Scaramucci

**Affiliations:** 1 Department of Restorative Dentistry, University of São Paulo, School of Dentistry, São Paulo, SP, Brazil.; 2 Department of Restorative, Preventive and Pediatric Dentistry, University of Bern. Bern, Switzerland

**Keywords:** tooth erosion, erosive tooth wear, desensitizing agents, fluoride, optical profilometry

## Abstract

This study evaluated the effect of actives present in conventional and desensitizing mouth rinses on the control of dentin erosive tooth wear. Two hundred and seventy dentin specimens from human molars were prepared. The specimens were randomly allocated into 10 experimental groups (n=10): 4 corresponding to desensitizing mouth rinses, 4 to conventional mouth rinses, a negative control group (C-: distilled water), and a positive control group (C+: 500 ppm fluoride plus 800 ppm tin mouth rinse). Specimens were subjected to an erosion-abrasion cycling model: 5 min immersion in 0.3% citric acid and 60 min exposure to artificial saliva. This procedure was repeated 4x/day for 5 days. Immediately after the first and last erosive challenges, the specimens were brushed with a slurry of fluoride toothpaste for 15 s, with a total of 2 min exposure to the slurry. Afterward, the specimens were exposed to the mouth rinses. Dentin surface loss (SL, in µm) was determined by optical profilometry. Data were statistically analyzed by using Kruskal-Wallis and Dunn's tests (α=0.05). The only mouth rinses that presented significantly lower dentin SL than the C- was a desensitizing one containing sodium fluoride (220 ppm F^-^) with dipotassium oxalate (1.4%) and the C+ (p<0.001 and p=0.013, respectively), without significant differences between them (p>0.05). Dentin SL of the other groups did not significantly differ from the C- (p>0.05). The combination of sodium fluoride with dipotassium oxalate in a desensitizing mouth rinse showed a promising result against dentin erosive wear, matching the protection offered by a fluoride/tin mouth rinse.

## Resumo

O presente estudo avaliou o efeito de ativos presentes em enxaguatórios convencionais e dessensibilizantes no controle do desgaste dentário erosivo na dentina. Duzentos e setenta espécimes de dentina, de molares humanos, foram preparados. Os espécimes foram randomizados e alocados nos 10 grupos experimentais (n=10): sendo 4 correspondentes aos enxaguatórios dessensibilizantes, 4 enxaguatórios convencionais, um controle negativo (C-: água destilada), e um controle positivo (C+: enxaguatório com 500ppm de flúor mais 800ppm de estanho). Os espécimes foram submetidos a uma ciclagem erosiva-abrasiva, sendo: 5 min de imersão em ácido cítrico a 0,3%, seguido de remineralização em saliva artificial, por 60 min. Esse procedimento foi repetido 4x/dia durante 5 dias. Imediatamente após o primeiro e o último desafio erosivo, os espécimes foram escovados com uma suspensão de dentifrício fluoretado por 15 s, com total de 2 min de exposição à suspensão. Em seguida, os espécimes foram expostos ao enxaguatório, de acordo com cada grupo experimental. A perda de superfície (PS, em µm) em dentina foi determinada por perfilometria óptica. Os dados foram analisados estatisticamente utilizando os testes de Kruskal-Walis e Dunn (α=0,05). O único enxaguatório que apresentou PS estatisticamente significante menor que o C- foi o dessensibilizante contendo fluoreto de sódio (200ppm de F-) e oxalato dipotássio (1,4%) e o C+ (p<0,001 e p=0,013, respectivamente), sem diferença estatística significante entre eles (p>0,05). A diferença de PS dos outros grupos não foi estatisticamente significativa, quando comparados ao C- (p>0,05). A combinação de fluoreto de sódio com oxalato dipotássio no enxaguatório dessensibilizante demonstrou um resultado promissor contra o desgaste dentário erosivo na dentina, sendo esta uma proteção similar à oferecida pelo enxaguatório contendo flúor e estanho.

## Introduction

Erosive tooth wear (ETW) is a condition of growing interest, with high prevalence rates reported, which have been associated with changes in eating and behavioral habits among populations ^1^. ETW is a chemical-mechanical process that occurs when erosive acids repeatedly contact the dental hard tissues, making this eroded surface more vulnerable to being removed by mechanical impacts ^2^. After the complete loss of enamel or cementum, dentin is affected, as acids are also capable of opening and increasing the diameter of the dentinal tubules, thus resulting in dentin hypersensitivity (DH) ^3^.

Fluoride is one of the most recommended agents for preventing and controlling ETW and DH. It can induce the precipitation of fluoride deposits on the tooth surfaces. These compounds are thought to offer surface protection against erosive acids ^4^, and to precipitate inside the dentin tubules, thus reducing DH ^5^. For an optimized action against DH, fluoride has been associated with other active ingredients, such as calcium sodium phosphosilicate (CSP), arginine combined with the copolymer polyvinylmethyl ether-maleic acid (PVM/MA), pyrophosphate, and dipotassium oxalate, as they could block the dentin tubules more effectively ^6^ ^,^ ^7^. Moreover, some of them were found to be acid-resistant ^7^. This dual action could be important because ETW is often associated with DH ^8^.

Desensitizing agents are commonly found in dentifrices, but currently, several mouth rinses claim to reduce DH (desensitizing mouth rinses) ^9^. However, the action of these products on the prevention of ETW has not been well explored so far. Desensitizing mouth rinses are easy and safe to be used without supervision, with the advantages of having a simpler composition and not involving the mechanical action of tooth brushing.

This is relevant in the context of ETW prevention because, in some cases, tooth brushing can reduce the protective effect of the active ingredients ^10^. Considering the close relationship between ETW and DH, and the importance of protecting the dentin against further tissue loss, it is relevant to evaluate whether the active agents present in desensitizing mouth rinses can also help to prevent ETW. Furthermore, investigating the protective effect of conventional mouth rinses against dentin erosive wear is also important, particularly given the limited protection offered to dentin by fluoride solutions ^11^.

In view of this, we aimed to evaluate *in vitro* the effect of actives in desensitizing and conventional mouth rinses on controlling dentin ETW. The null hypothesis of the study was that dentin surface loss would not differ among mouth rinses when tested in an erosion-abrasion cycling model.

## Materials and Methods

### Experimental Design

This study followed a completely randomized design with one experimental factor: treatment, at 10 levels, corresponding to the mouth rinses. Table 1 shows the description of the experimental groups. The mouth rinses were tested using a 5-day erosion-abrasion cycling model. For this purpose, dentin specimens obtained from human molars were used (n=10). The response variable was dentin surface loss (µm) measured by optical profilometry at the end of the cycling.

### Sample size calculation

The sample size calculation was based on a pilot study, and was performed using the ANOVA Sample Size Test, with the software SigmaPlot 13 (Systat Software Inc, San Jose, CA, USA). Considering an effect size of 4.21, a standard deviation of 1.4 for surface loss, α=0.05 and a power of 0.80, we obtained a sample size of 5. Considering this and other investigations in field, we adopted n=10.

### Specimen preparation

In the present study, sound human molars were used after obtaining approval from the Local Ethics Committee on Research with Human Beings (CAAE number: 89906318.7.0000.0075). The teeth were previously stored in 0.1% of thymol solution (Sigma-Aldrich, St Louis, MO, USA), under refrigeration at 4˚C, until the beginning of the experiment. From the roots of the teeth, 270 dentin fragments (3 mm x 3 mm x 2 mm) were obtained using an automatic cutting machine (Isomet 1000 Precision Saw, Buehler, Lake Bluff, IL, USA). The specimens were ground flat and polished with abrasive papers of decreasing grain size (800, 1200, and 4000 grit; Struers Inc.) in a polishing machine (Tegramin, Struers Inc., Ballerup, Denmark) under constant cooling. Between the different paper grits and after the final polishing, the specimens were sonicated with distilled water for 3 min to remove any debris. After polishing, specimens were analyzed under a stereoscopic microscope to discard the ones with cracks or any other structural defects ^9^.

### Analysis of the initial curvature of the specimens

A central area of 2 mm x 1 mm on all specimens was analyzed with an optical profilometer (Proscan 2100, Scantron Ltd, Venture Way, Taunton, UK), as described in the “Profilometric analysis” section below. Specimens with initial curvature values higher than 0.3 µm were discarded ^11^. The suitable specimens had two-thirds of their surfaces protected with adhesive tape to create two reference areas, leaving a central area (3 mm x 1 mm) exposed to the following challenges.

### EDTA Conditioning - open the dentinal tubules and treatments.

To simulate hypersensitive dentin, the condition that would require the use of a desensitizing mouth rinse, the dentin tubules were opened by immersing the specimens in EDTA solution (17%, pH=7.4) for 5 min ^12^. After this, the specimens were abundantly rinsed with distilled water. Subsequently, they were randomly allocated into the 10 experimental groups (Table 1), n=10.

**Table 1:** Experimental groups, their codes, mouth rinses composition, the protocol of application, and pH values.

Group	Product	Manufacturer	Main Composition	Protocol (immersion time)	pH
Desensitizing mouth rinses					
G1	Colgate^â^ Sensitive Pró-Alívio^TM^	Colgate Palmolive Industrial Ltda, Brazil	Arginine (0.8%), Aqua, Glycerin, Sorbitol, Propylene Glycol, Tetrapotassium Pyrophosphate, PEG-40, Hydrogenated Castor Oil, PVM/MA Copolymer, Polysorbate 20, Tetrasodium Pyrophosphate, Aroma, Benzyl Alcohol, Sodium Fluoride (NaF) (225ppm F^-^), Menthol, Sodium Saccharin, Citric Acid, Methylisothiazolinone, Cl 42051, Cl 17200	60 s	8.38
G2	elmex^â^ Sensitive Professional^TM^	GABA, Colgate Palmolive, Swidnica	Arginine (0.8%), Aqua, Glycerin, Sorbitol, Propylene Glycol, Disodium Pyrophosphate, PEG-40 Hydrogenated Castor Oil, PVM/MA Copolymer, Tetrapotassium Pyrophosphate, Sodium Levulinate, Olaflur (AmF) (125 ppm F^-^), Aroma, Potassium Hydroxide, Sodium Saccharin, Sodium Fluoride (NaF) (125 ppm F^-^), C.I. 19140, C.I. 42051	30 s	6.23
G3	Listerine^â^ Advanced Defence Sensitive	Johnson & Johnson Limited Maidenhead, UK.	Dipotassium Oxalate (1.4%), Aqua, Sorbitol, Propylene Glycol, Phosphoric Acid, Aroma, Poloxamer 407, Sodium Benzoate, Sodium Methyl Cocoyl Taurate, Sodium Lauryl Sulfate, Sucralose, Sodium Saccharin, Sodium Fluoride (NaF) (220 ppm F^-^)	60 s	4.24
G4	Sensodyne^â^ Cool Mint	Glaxosmithkline Brasil Ltda, Jacarepaguá, RJ, Brazil	Aqua, Glycerin, Sorbitol, Potassium Nitrate, PEG-60 Hydrogenated Castor Oil, Poloxamer 407, Sodium Benzoate, Aroma, Disodium Phosphate, Methylparaben, Propylparaben, Sodium Phosphate, Sodium Fluoride, Sodium Saccharin, CI 42090. Contains 3% w/w Potassium Nitrate and 0.048% w/w Sodium Fluoride (NaF) (217 ppm F^-^)	60 s	6.65
Conventional mouth rinses					
G5	Colgate^â^ Plax Soft Mint	Colgate Palmolive Industrial Ltda, Brazil	Aqua, Glycerin, Propylene Glycol, Sorbitol, Poloxamer 407, Aroma, Cetylpyridinium Chloride, Potassium Sorbate, Sodium Fluoride, Sodium Saccharin, Menthol, CI 42051, Contains: Sodium Fluoride (NaF) 0.05% (225 ppmF^-^)	60 s	5.09
G6	elmex^®^ Caries	GABA, Colgate Palmolive, Swidnica	Aqua, Propylene Glycol, PEG-40 Hydrogenated Castor Oil, Olaflur, Glycerin, Sodium Benzoate, Levulinic Acid, Sodium Levulinate, Aroma, Saccharin, Sodium Fluoride, Sodium Anisate. Contains: Olaflur (AmF) and Sodium Fluoride (NaF) total fluoride content: 250 ppm. Aroma (> 100 PPM): Anethole, Menthol	30 s	4.45
G7	Listerine^®^ Anticaries^TM^	Johnson & Johnson Brazil. Industry and Trade of Health Products Ltda	Aqua, Sorbitol, Propylene Glycol, Poloxamer 407, Sodium Lauryl Sulfate, Benzoic Acid, Aroma (Benzyl Alcohol, d-limonene), Eucalyptol, Methyl Salicylate, Thymol, Sodium Saccharin, NaF (220 ppm F^-^), Sodium Benzoate, Sucralose, Menthol, CI 47005, CI 42053	60 s	4.18
G8	Pronamel^®^ Daily Mouthwash	Glaxosmithkline group of companies, UK	Aqua, Glycerin, Sorbitol, Poloxamer 338, PEG-60 Hydrogenated Castor Oil, VP/VA Copolymer, Potassium nitrate, Sodium benzoate, Cellulose Gum, Aroma, Sodium fluoride, Methylparaben, Propylparaben, Cetylpyridinium Chloride, Sodium Saccharin, Xanthan Gum, Disodium Phosphate, Sodium Phosphate, CI 42090, NaF 0.1% w/w (450 ppm F^-^)	60 s	6.29
Controls					
Negative Control	Distilled water	-	Distilled water	30 s	4.5
Positive Control	Elmex Erosion Protection		Aqua, Glycerin, Sodium Gluconate, PEG-40 Hydrogenated Castor Oil, Aroma, Stannous Chloride (800 ppm Sn^2+^), Cocamidopropyl,Betaine, Sodium Saccharin, Sodium Fluoride and Olaflur (Aminofluorid) (500 ppm F^-^)		

### Erosive-abrasive cycling

The erosive-abrasive cycling was conducted following previous studies ^13^ ^,^ ^14^. The specimens were immersed in citric acid (0.3%, pH=2.6) for 5 min, followed by immersion in artificial saliva (0.213 g/l CaCl_2_·2H_2_O; 0.738 g/l KH_2_PO_4_; 1.114 g/l KCl; 0.381 g/l NaCl; 12 g/l Tris buffer, pH adjusted to 7.0 with KOH) ^13^ for 60 min. This procedure was repeated 4 times/day for 5 days ^14^. Immediately after the first and the last erosive challenges, the specimens were brushed in an automatic brushing machine (Biopdi, Sao Paulo, Brazil) for 15 s (150 g, 45 strokes). Brushing was performed with soft brushes (Oral-B Indicator Plus, Procter & Gamble, São Paulo, Brazil) and a toothpaste slurry, which was prepared by mixing a conventional fluoride toothpaste (Elmex Anticáries, Colgate Palmolive, Brazil, 1400 ppm of F^-^, as amine fluoride) with artificial saliva, in a ratio of 1 part of toothpaste to 3 parts of artificial saliva. The slurry was prepared before each abrasive challenge and the specimens were exposed to toothpaste slurries for a total of 2 min. Afterward, the specimens were exposed to the mouth rinses for 30 s or 60 s, depending on the experimental group (Table 1). After each erosive and abrasive challenge, the specimens were rinsed with distilled water and gently dried with absorbent paper. At the end of each cycling day, the specimens were kept in a humid chamber overnight ^13^. The citric acid solution and mouth rinses were replaced after each use, and artificial saliva was renewed once a day, at the beginning of the cycle.

### Profilometric Analysis

At the end of the cycling, the adhesive tapes were removed from the surfaces, and the specimens were kept in a humid chamber at room temperature before the profilometric analysis ^11^. The instrument sensor scanned an area 2 mm long (x-axis) by 1 mm wide (y-axis), located at the center of the specimen. The equipment was set to go through 200 steps on the x-axis, with each step measuring 0.01 mm. On the y-axis, there were 20 steps measuring 0.05 mm each. Using specific software (Proscan Application software version 2.0.17, Scantron Ltd), the dentin surface loss was calculated by subtracting the average height of the exposed area from the average height of the reference areas. The results were expressed in micrometers (μm).

### Statistical analysis

Normality and homoscedasticity of the data were checked with Shapiro-Wilks and Brown-Forsythe tests, respectively. Since data did not follow a normal distribution, comparisons between groups were made using Kruskal-Wallis and Dunn tests, with a significance level of 5%. The software SigmaPlot 13 (Systat Software Inc, San Jose, CA, USA) was used for the analysis.

## Results

**Figure 1:**
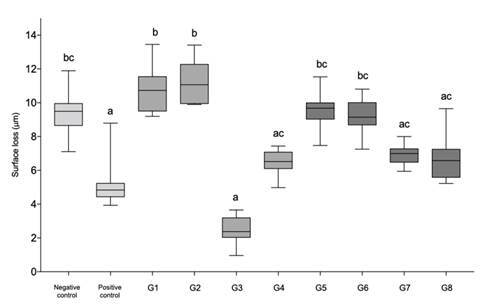
Medians and interquartile ranges for dentin surface loss for all groups after erosion-abrasion cycling. Different letters denote significant differences between groups (p<0.05)

The medians and interquartile ranges for dentin surface loss of all groups after erosive-abrasive cycling are shown in Figure 1. The only mouth rinse that showed significantly lower surface loss than the negative control was the one containing dipotassium oxalate and fluoride (G3) and the positive control (p<0.001 and p=0.013, respectively), with no significant differences between them (p>0.05). The other groups did not differ significantly from the negative control (p>0.05); however, both desensitizing mouth rinses containing arginine, PVM/MA, pyrophosphate, and fluoride (G1 and G2) exhibited significantly higher dentin loss values than G3, positive control, G4, G8 and G7 (p > 0.05).

## Discussion

In the present study, significant differences were observed in dentin surface loss between the mouth rinses after the erosion-abrasion cycling, thus our null hypothesis was rejected. The mouth rinse that showed the lowest dentin surface loss was a desensitizing one containing 220 ppm of F^-^, as sodium fluoride, and 1.4% of dipotassium oxalate (G3). Dipotassium oxalate can react with the ionized calcium on dentin, forming insoluble calcium oxalate crystals that could seal the dentin tubules and reduce the hydraulic conductance of dentin ^15^. In our study, the repeated applications of the mouth rinse during the cycling may have allowed the formation of a more coherent layer of calcium oxalate monohydrate in the subsurface crystalline region, which was resistant to several erosive and abrasive challenges. Consistent with this, it was shown that this mouth rinse had features of subsurface occlusion that were least affected by an erosive challenge ^16^. The dipotassium oxalate mouth rinse could provide more stable tubule occlusion, being better able to withstand an acid challenge compared with a mouth rinse that contained arginine, PVM/MA copolymer, and pyrophosphate ^7^. This latter mouth rinse reduced dentin permeability by 32% versus nearly 100% reduction for the mouth rinse containing dipotassium oxalate after 12 treatments. Although evaluating the action of the active ingredients in reducing dentin permeability was not the aim of this study, it is important to note that there is a close relationship between ETW and DH, so a reduction in tubule patency could also be associated with less acid diffusion within the dentin. In addition to fluoride, the mouth rinses from groups G1 and G2 contain arginine, PVM/MA copolymer and pyrophosphates as active ingredients. In vitro studies have suggested the formation of a layer consisting of arginine and PVM/MA copolymer on the dentin surface after their application ^6^. The copolymer PVM/MA has bioadhesive properties, which could allow the formation of carboxylic groups from the polyanhydride residues, thereby favoring the formation of hydrogen bonds between the copolymer and the dentin surface ^17^. The combination of PVM/MA copolymer, arginine, and pyrophosphate salts relies on forming an adhesive complex on the dentin surface, which allegedly builds up after repeated applications. As a result, a reduction in dentin permeability could be expected ^6^. However, in our study, the mouth rinses containing these compounds were not able to protect the dentin against erosive-abrasive challenges; on the contrary, they demonstrated the highest values of dentin surface loss. Indeed, the surface loss values of these mouth rinses did not differ significantly from those of the negative control group or from their conventional versions that do not contain arginine or copolymer (G5 and G6). It could be suggested that the protective layer formed with the use of these desensitizing mouth rinses was not stable enough to withstand the effect of multiple erosive and abrasive challenges.

The other mouth rinses tested (G5, G7 and G8) showed intermediate results, and did not differ significantly from the negative control group or G4. For G8, this was unexpected since it has a higher fluoride content. Most of the mouth rinses tested in the present study had a fluoride concentration in the range of 217 to 250 ppm, except for G8 and the positive control, which have concentrations of 450 ppm F^-^and 500 ppm F^-^, respectively. Besides fluoride concentration, low pH is also a factor that can favor the deposition of CaF_2_-like material on the tooth surfaces ^18^. The pH of G8 is 6.29, much higher than that of the positive control rinse (4.47) and the dipotassium oxalate rinse (4.24). Nonetheless, previous studies have shown that, even though some formulations allow the deposition of higher amounts of adsorbed fluoride, this is not always translated into improved protection against ETW ^13^ ^,^ ^19^. From our results, similarly to what was observed for enamel ^4^, it could be suggested that the effect of conventional and desensitizing mouth rinses containing monovalent fluoride compounds on dentin ETW is minimal unless other ingredients in their formula offer additional protection, as tin or dipotassium oxalate.

The mouth rinse chosen as the positive control is specifically recommended for treating ETW and contains both fluoride and tin. According to a systematic review, the combination of fluoride and tin in mouth rinses provides higher protection against enamel erosion and ETW than fluoride alone, whose effect is minimal or nonexistent ^4^. Evidence from in situ studies also supports greaterr benefits with the use of F/Sn mouth rinse for dentin ^21^ ^,^ ^22^. In dentin, tin can deposit as different mineral compounds at the surface, such as Sn_3_F_3_PO_4_ and Sn_2_OHPO_4_ ^23^, or incorporate into its structure under cyclic conditions. This effect is mainly observed when the dentin collagen matrix is retained ^23^, which usually occurs under in vitro conditions ^24^ . Under clinical conditions, however, the organic matrix is thought to be degraded by enzymatic action, potentially impacting the protective effect of the components ^24^. There is only limited clinical data about the efficacy of F/Sn mouth rinses on ETW ^25^. Nonetheless, an *in situ* investigation has shown that when used once a day, the F/Sn mouth rinse can reduce dentin erosion by about 47% compared to the control ^22^. A similar level of reduction (approximately 49%) was observed in another in situ study, but the F/Sn rinse was applied twice daily ^21^. One drawback of regularly using tin compounds is tooth discoloration ^25^. Therefore, finding new agents to improve fluoride’s effect against ETW is still desirable.

One limitation of the present in vitro investigation is that, clinically, the mouth rinse is swished around the oral cavity, allowing fluoride retention at other sites, such as the soft oral tissues ^7^, and this was not fully reproduced here. Additionally, when introduced into the mouth, the rinses become diluted by saliva and interact with the salivary pellicle at the tooth surfaces. All these factors should be considered when extrapolating our findings. Another limitation is that no microscopic images were obtained to qualitatively evaluate the surface of the specimens, although these images could be assessed in a previous study from our group that tested these desensitizing mouth rinses for their ability to promote tubule occlusion ^9^. The erosive cycling performed was based on previous investigations ^11^ ^,^ ^14^ ^,^ ^20^ and we opted for a 5 min erosive challenge because this is within the time frame that intraoral pH remains acidic after consuming an acid drink ^26^. Although the erosion-abrasion model performed may be considered aggressive, it should be noted that most *in vitro* and *in situ* ETW studies employ accelerated erosion/abrasion conditions, as ETW is a condition that typically takes years to develop. This approach allows laboratory studies on ETW to be conducted feasibly. Additionally, it has been observed that more aggressive erosion conditions can better differentiate the effect of the treatments, especially for fluoride rinses ^27^. Nonetheless, it could be speculated that if a milder erosive challenge had been adopted, some other mouthrinse might have shown a protective effect. The surface loss values obtained in this study are within the range found in previous studies testing mouthrinse against erosion and using profilometry as the response variable ^20^ ^,^ ^21^.

In conclusion, the only mouth rinse that significantly reduced dentin surface loss compared to the negative control was the one containing dipotassium oxalate and sodium fluoride, which matched the protection offered by the combination of fluoride and tin mouth rinse. In this context, dipotassium oxalate stands out as a new agent to be further evaluated against ETW in combination with fluoride.
